# Graftless sinus augmentation technique with contextual placement of implants: a case report

**DOI:** 10.1186/1752-1947-8-437

**Published:** 2014-12-17

**Authors:** Nicolae Chipaila, Roberta Marini, Gian Luca Sfasciotti, Alessandro Cielo, Laura Bonanome, Annalisa Monaco

**Affiliations:** 33 University of L’Aquila, Department of Life, Health and Environmental Sciences, Unit of Dentistry, Delta 6 building, Via Vetoio, 1, 67100 L’Aquila, Italy; 34 ‘Sapienza’ University of Rome - Department of Oral and Maxillofacial Sciences, Via Caserta, 6, 00161 Rome, Italy; 35 ‘Private Practice, Via Regina Elena 287/A, 00161 Rome, Italy

**Keywords:** Sinus augmentation, Maxillary alveolar ridge atrophy, Implant placement

## Abstract

**Introduction:**

The positioning of implants in the jaw bones with contextual graftless lateral approach sinus lifting is finding an increasingly broad consensus in the literature.

Since the 1970s, various clinical research projects have been conducted on applications of biological and synthetic biomaterials in bone regenerative surgery, both in sinus lift procedures and in cystic cavity filling after cystectomy or in bone defects in regenerative periodontal surgery. Currently, we are finding that there is an increasing trend of clinicians aiming to adopt graftless techniques, with satisfactory results in terms of implant survival in the long term.

In our study, through a case report, we describe a variant of graftless sinus augmentation technique with contextual implant placement, emphasizing the role of the blood clot, combined with collagen sponges, as a natural scaffold and the osteogenic potential of the subantral membrane in guided bone regeneration, with reduced morbidity of the patient.

**Case presentation:**

To describe the surgical technique, the clinical case of a 38-year-old Caucasian woman with a lateral posterior edentulism was selected. The rehabilitation was solved by a graftless sinus augmentation technique with a contextual implant placement.

For each implant, a resonance frequency analysis evaluation was reported as implant stability quotient values. The performance of the implant stability quotient values followed a gradual increase from time zero to the sixth month, as the clot was differentiated into osteoid tissue and then into bone tissue, due to the scaffold effect conferred by the equine collagen sponge. The stabilization phase took place between the fourth and the sixth month, according to the implant stability quotient values.

**Conclusions:**

Our graftless sinus augmentation technique seems to be very predictable thanks to the osteoconductive principles on which it is based, and in association with the proper management of peri-implant soft tissue, so as to increase the amount of keratinized tissue, which could represent the new gold standard for this type of rehabilitation in the future.

## Introduction

### Maxillary sinus augmentation procedures’ background

Oral implantology in prosthetic rehabilitation is certainly one of the most important acquisitions for dentistry in the last sixty years.

Often, in the posterior region of maxilla, following the loss of teeth, there is a reduction in volume of the bone crest and this reduction is further exacerbated by increased pneumatization of the maxillary sinuses. Therefore, the presence of a residual bone crest volumetrically unsuitable for the insertion of endosseous implants has led to the definition of surgical procedures designed to restore a suitable bone volume for implant procedures.

The maxillary sinus augmentation procedure is currently considered a highly predictable and safe technique that allows the insertion of osseointegrated implants into the atrophic posterior maxilla [1], where the pneumatization of Higmoro’s antrum on one side and the edentulous alveolar process resorption by another side, often compete to make implant anchorage prosthetic rehabilitation impossible [2–7].

At the end of the 1960s, Linkow [8] first referred to the possibility of introducing blade implants into the maxillary sinus, by partially lifting the Schneider membrane without tearing it.

In 1975, Tatum proposed to raise the sinus membrane by performing a modified Caldwell-Luc technique, then called the ‘inverted lateral window’, introducing, as graft, autologous bone taken from the rib [9].

The first publication of this technique is due to Boyne and James [10], who in 1980 reported 14 cases of autologous graft (iliac crest) with simultaneous insertion of blade implants. Branemark [11] in 1984 reported 139 penetrating implants into the maxillary sinus and nasal cavity with a follow-up from two to ten years.

In 1986, Smiler and Holmes [12] proposed a technique that involves the use of nonresorbable hydroxyapatite as a bone graft substitute.

Several grafts are currently used: autologous, homologous, heterologous or alloplastic. Moy [13] and Smiler [14] have published studies in which the different materials are compared.

In 1987, Misch [15] proposed a maxillary atrophy classification that also takes into account the therapeutic solution, as well as the most recent classification proposed by Favero and Branemark [16] in 1994: it takes into account the maxillary atrophy in its entirety.

There is currently no single protocol to follow when planning this type of surgical operation: some variables such as the crestal height, the separation between the walls and the sinus pneumatization, the state of the membrane or the type of residual bone in the crest, affect the surgical indication and the techniques that must be adopted.

Antroplastic techniques can be divided into: maxillary sinus augmentation procedures (or lateral access sinus lifts) and mini sinus lifts (or crestal access sinus lifts). The first are those most frequently indicated in large-volume bone regeneration, the second are indicated in lower-volume regeneration, taking advantage of the preparation of the implant site such as access to the maxillary sinus.

The lateral access technique with insertion of various types of graft has been well codified and showed a good success predictability of the grafts and implants inserted. In the last 30 years it has undergone numerous changes, aimed at reducing the overall invasive surgery, the intra- and postoperative complications, and the patient’s morbidity.

The excessive opening of the side window, the extraoral bone graft harvesting and the late implant insertion made the surgical operation invasive and expensive.

Initially, the surgical technique used for antrostomy access made use of rotary instruments and the most frequent intraoperative complication was the perforation of the sinus membrane, with percentages ranging between 20 and 30% for different authors [17, 18].

Lately, oral surgeons have begun to use a piezoelectric tool to perform the antrostomy and the percentage of perforations has significantly decreased [19, 20].

The crestal access, on the other hand, involves less surgical invasiveness and has an equally predictable success rate of endosseous implants.

The crestal approach sinus-lifting technique introduction has further increased the indications for contextual implant placement and alveolar ridge increase. In these cases, the technique requires dedicated instrumentation and remarkable operator sensitivity during the lifting of the membrane to avoid tearing. In any case, the indication to perform a crestal approach with simultaneous implant insertion appears to be limited to clinical situations with residual alveolar ridge height >5mm and where the requested vertical increase does not exceed 5mm [21, 22].

In addition to the surgical techniques, the interest of clinical research has focused particularly on the biomaterials used to perform the filling of the lifted maxillary sinus [21], as the ability to generate bone in its interior was attributed mainly to the biomaterials’ intrinsic characteristics rather than the spontaneous healing capacity of the area [23].

The biomaterials used nowadays can be divided into autogenous (derived from the same patient), allogenic (obtained from another human being), and xenogenic (derived from another animal species).

Autologous bone is the gold standard for its recognized inductive and conductive abilities and because of its intrinsic osteogenicity [24]. It, in fact, coming from the same patient, guarantees the complete absence of adverse immune response.

Mineralized or demineralized freeze-dried alloplastic bone grafts (FDBA, DFDBA), xenografts of bovine origin, sulphate and calcium phosphate, hydroxyapatite, and bioglass have been widely employed and scientifically evaluated in order to determine the formation of new bone within the maxillary sinus and to allow osseointegration of the implants.

All substitutes possess the ability to form bone even if there is a wide range of results from a histomorphometric point of view [25–28].

Bone regeneration, in fact, follows valid principles [29, 30] independently of the type of graft used, according to which there is the possibility of new bone formation whenever it shall create a space that can be maintained, favoring growth factors input and avoiding infectious phenomena.

In this report, a graftless sinus augmentation technique with contextual implant placement is described: the use of a simple collagen sponge is able to stabilize the blood clot in the early stages of healing.

### Blood clot and Schneiderian membrane osteogenic potential

The blood clot regenerative potential is currently a topic of much discussion in the literature and it is the research subject of many authors, both for its application in guided bone regeneration (GBR) and for its application in graftless sinus augmentation techniques [6, 7, 31–33].

The blood clot revaluation, as the only filler in the maxillary sinus augmentation technique in an era in which the biological and synthetic biomaterials seem to have taken over, is mainly due to Lundgren, who observed, after a maxillary sinus cyst enucleation, that the cavity left by the membrane elevation after the cyst removal was filled with bone within three months without doing anything.

In this context, he decided to further investigate this phenomenon by developing a new surgical approach, which consisted of the following steps: to carve the bone window with a beveled incision and then to remove it completely, to raise the Schneiderian membrane without tearing it, to insert the implant fixtures and to relocate the bone operculum, allowing the blood clot to fill the free space between the sinusal membrane and basal bone. The histological analysis confirms the perfectly vital bone formation starting from the surface of the antral membrane, so assuming its osteogenic potential [31, 34, 35].

Lambert *et al.* in 2010 [36], compared, in a study on rabbits, different materials to be used as fillers in the function of subantral bone regeneration, including the blood clot, autogenous bone and bovine hydroxyapatite (BHA): all three space-fillers allowed bone formation. The authors emphasized that the blood clot is an excellent growth factor carrier, showing initially a faster and greater bone formation, but the increase in volume is significantly reduced at five weeks postoperatively, showing that the blood clot alone may not be able to provide an adequate resistance to the sinusal re-expansion.

These observations are in agreement with other authors [37, 38], who stress that the osteoinductive properties of the blood clot alone would, therefore, be limited primarily by the inability to maintain the created space.

In this context, in our report we have considered the importance of maintaining blood clot stability through the simultaneous insertion of the implant fixtures and the association of the collagen sponge, in order to facilitate the membrane repositioning maintenance in the long term.

The results, assessed clinically and radiographically, confirmed the osteoinductive effects of the blood clot, and did not find any limitations in relation to the difficulty of maintaining the created space, overcome by the strategy of our surgical technique.

Various studies have also been conducted on the Schneiderian membrane and on its osteogenic potential [32, 33].

Srouji *et al.* [32] have demonstrated both *in vitro* and *in vivo* that the antral membrane contains osteoprogenitor cells able to proliferate and differentiate: the authors have thereby provided a biological background for the understanding of the clinical phenomenon observed in the surgical procedure.

Histologically, the Schneider membrane is composed of several layers: an epithelial lining, a richly vascularized lamina propria, and a deeper layer that covers the jawbone. This last layer is the interface with the underlying bone, and could be compared to the periosteal structure [32].

*In vitro*, the osteoprogenitor cells in culture have been brought to secrete alkaline phosphatase, BMP-2, osteopontin, osteonectin, osteocalcin, and also to mineralize their extracellular matrix, as already demonstrated by Gruber *et al.* in 2004 [39] with cells of porcine sinus mucosa. *In vivo*, the heterotopic implantation of membrane cells combined with an osteoconductive scaffold led to the formation of new trabecular bone.

In a subsequent report, Srouji *et al.* [33] through an *in vivo* simulation of an animal model sinus lift, showed the osteogenic potential of Schneider’s membrane and its possible contribution to bone regeneration in sinus lift procedures.

Palma *et al.* [34] in a study on primates, inserted Brånemark implants both with a smooth and an oxidized surface, with a contextual sinus lift with or without autogenous bone application and analyzed the results at six months by performing block sections, reporting these conclusions:

1. the obtained bone augmentation amount was not significantly different between the group with an autogenous bone graft and the one without a graft;
2. the use of surface-treated implants improved the bone-implant contact;
3. new bone was evident between the Schneiderian membrane and the graftless implant sites, demonstrating therefore, the osteoinductive potential of the membrane.

These acquisitions bring further understanding to intimate biological mechanisms that are the basis of our study and confirm the trend to implement therapeutic strategies in which the body may be enabled to express the greatest potential for healing with the least possible external interference, increasing predictability and decreasing the potentially negative external variables.

### Aim of the study

In this report, we present the implant-prosthetic rehabilitation of a maxillary posterior edentulism case (area 1.4 to 1.5) through a graftless sinus augmentation technique and contextual implant fixtures placement. The purpose of the study is to describe the surgical technique aimed at the edentulous site implant rehabilitation, enhancing the role of the blood clot as a biofiller between implant screw and sinus membrane, and stressing the Schneiderian membrane osteogenic potential.

## Case presentation

**Figure 1:**
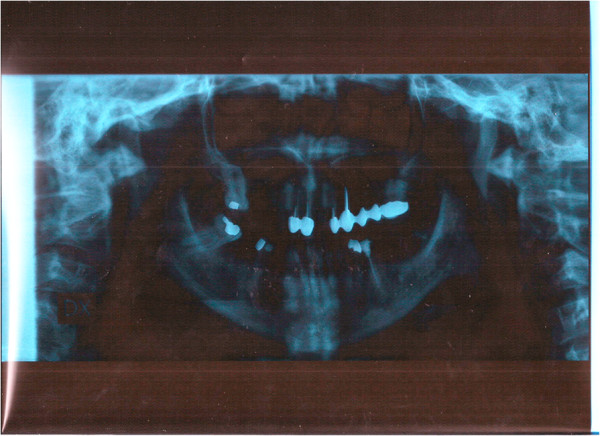
**Preoperative orthopanoramic X-ray.**

Our patient (a Caucasian woman, aged 38), with no history of previous or current diseases, presented with a maxillary lateral edentulism in the area 1.4 to 1.5. The preliminary radiographic evaluation showed bone dimensions of about 6 to 8mm in the coronoapical direction and 4 to 6mm in the bucco-oral direction (Figure 1).

**Figure 2:**
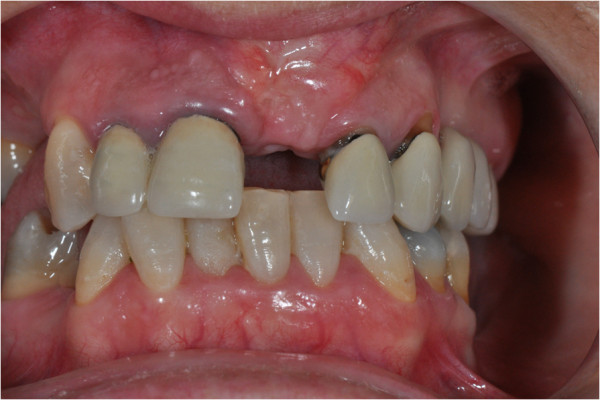
**Surgical procedure of sinus lift and implants placement.**

**Figure 3:**
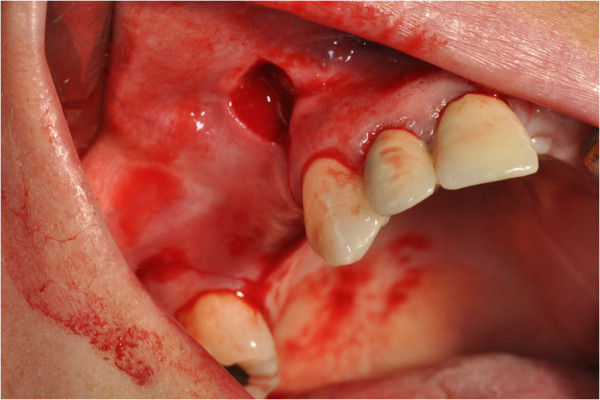
**Surgical procedure of sinus lift and implants placement.**

**Figure 4:**
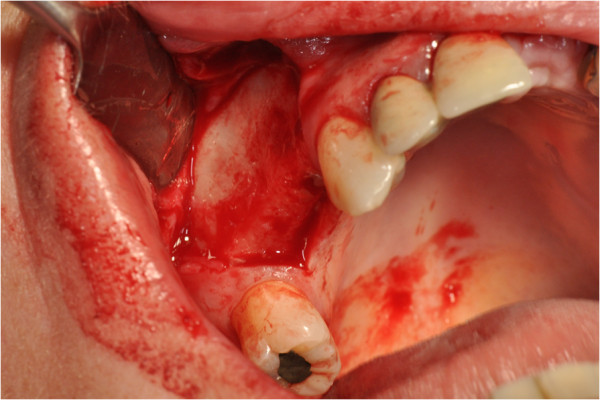
**Surgical procedure of sinus lift and implants placement.**

**Figure 5:**
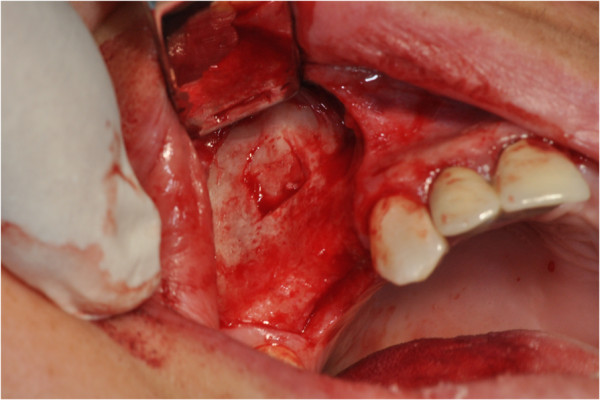
**Surgical procedure of sinus lift and implants placement.**

**Figure 6:**
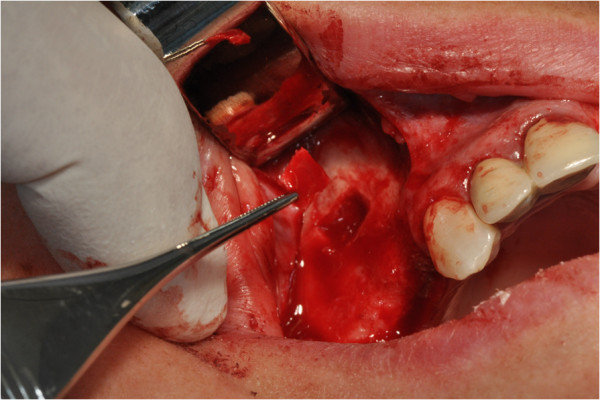
**Surgical procedure of sinus lift and implants placement.**

**Figure 7:**
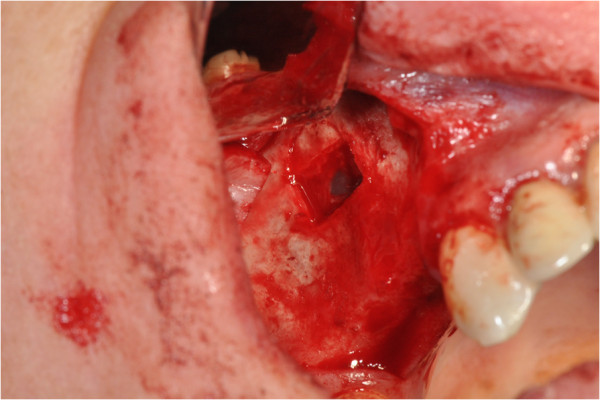
**Surgical procedure of sinus lift and implants placement.**

**Figure 8:**
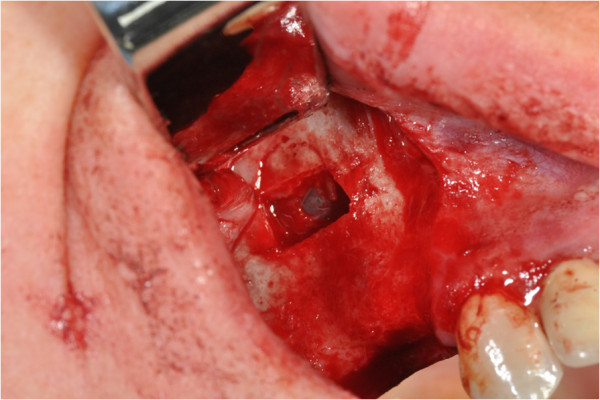
**Surgical procedure of sinus lift and implants placement.**

**Figure 9:**
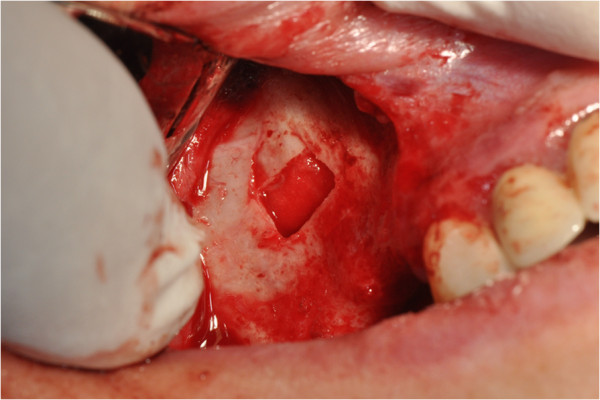
**Surgical procedure of sinus lift and implants placement.**

**Figure 10:**
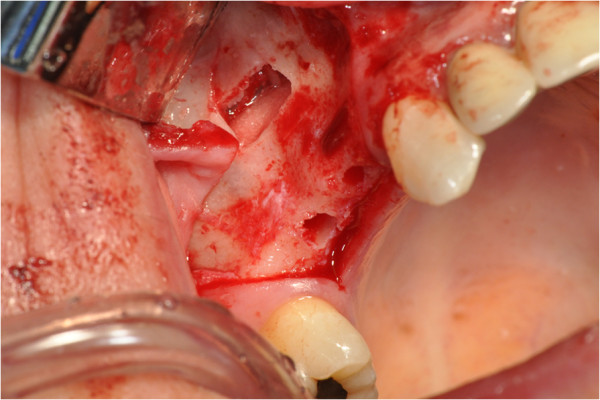
**Surgical procedure of sinus lift and implants placement.**

**Figure 11:**
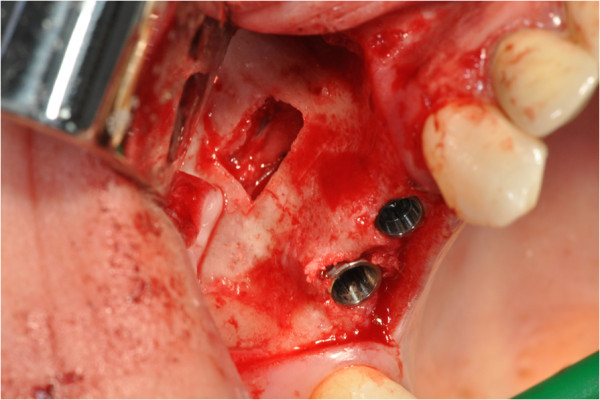
**Surgical procedure of sinus lift and implants placement.**

**Figure 12:**
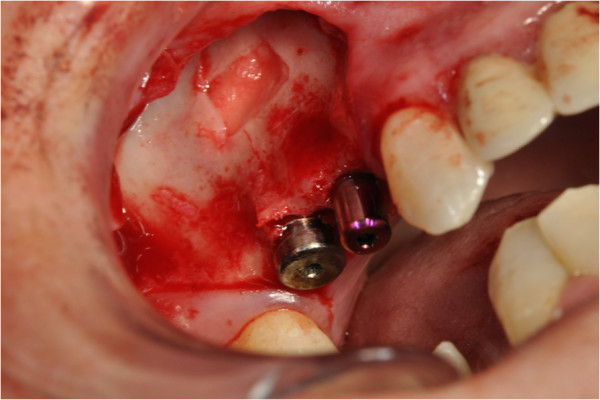
**Surgical procedure of sinus lift and implants placement.**

**Figure 13:**
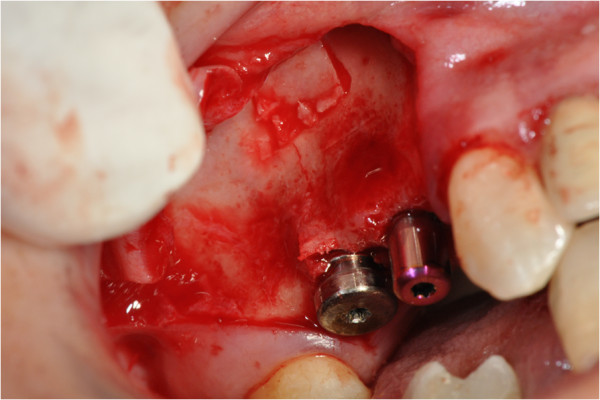
**Surgical procedure of sinus lift and implants placement.**

**Figure 14:**
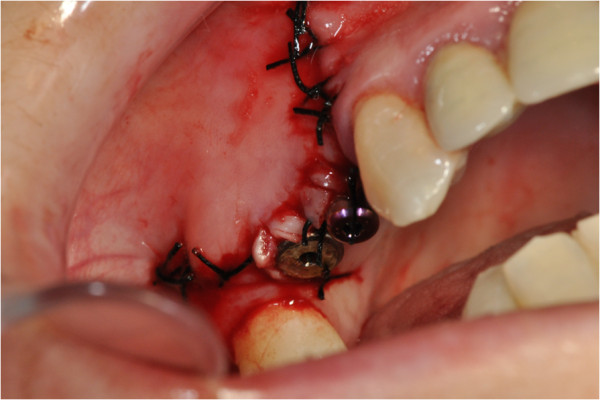
**Surgical procedure of sinus lift and implants placement.**

After the preliminary evaluation, the operation planning started. The rehabilitation of this maxillary edentulism was solved through the placement of two implant fixtures with the contextual sinus augmentation procedure (Figures 2, 3, 4, 5, 6, 7, 8, 9, 10, 11, 12, 13 and 14). The surgical operation was performed under local anesthesia, using mepivacaine (20mg/ml) with adrenaline (1:100,000), using the truncal technique at the infraorbital foramen and major palatine foramen. On the buccal side, a full-thickness paramarginal trapezial flap extended from element 1.3 to element 1.7 was performed. Subsequently, a lateral bony window with a Beaver 65 blade used as a surgical scalpel was made.

The bone segment was removed by anatomical tweezers and stored in sterile physiological solution at 4°C. The sinusal membrane was detached by special retractors and raised so as to achieve a curtain effect. An equine collagen sponge was placed in the antral area between the sinus floor and the raised membrane, to protect the latter under the Prichard’s vane ending, during the surgical alveoli preparation.

The neo-alveoli for the implant fixtures placement were performed through the crestal area, with the implant kit drills.

The two implants, with a diameter of respectively 3.5mm (normal platform, NP) and 4.3mm (regular platform, RP), a length of 13mm and conical connection characteristics were placed *in situ*. The primary stability was measured by resonance frequency analysis (RFA).

The apical area of the fixtures was soaked by the blood clot, in association with an equine collagen sponge, in the sinus area between the sinus floor and the raised membrane. The bone segment mobilized to perform the bone window was repositioned, the surgical area was covered with a collagen sponge layer and the flap was sutured with a nonabsorbable polyfilament.

After monitoring our patient for half an hour, no hemorrhage signs or local and/or general suffering was observed, so she was discharged with a prescription for antibiotic therapy (amoxicillin + clavulanic acid cpr. 1g for oral administration every 12 h for six days), anti-inflammatory (sodium naproxen cpr. 275mg for oral administration every 12 h for three days) and oral antiseptic therapy (0.2% chlorhexidine + cetilpiridine chloride).

Recommendations for oral hygiene and feeding techniques were supplied with the aim of optimizing the postoperative course.

The suture removal on the seventh day showed a good healing of tissues, with a modest share of keratinized tissue differentiation.

**Figure 15:**
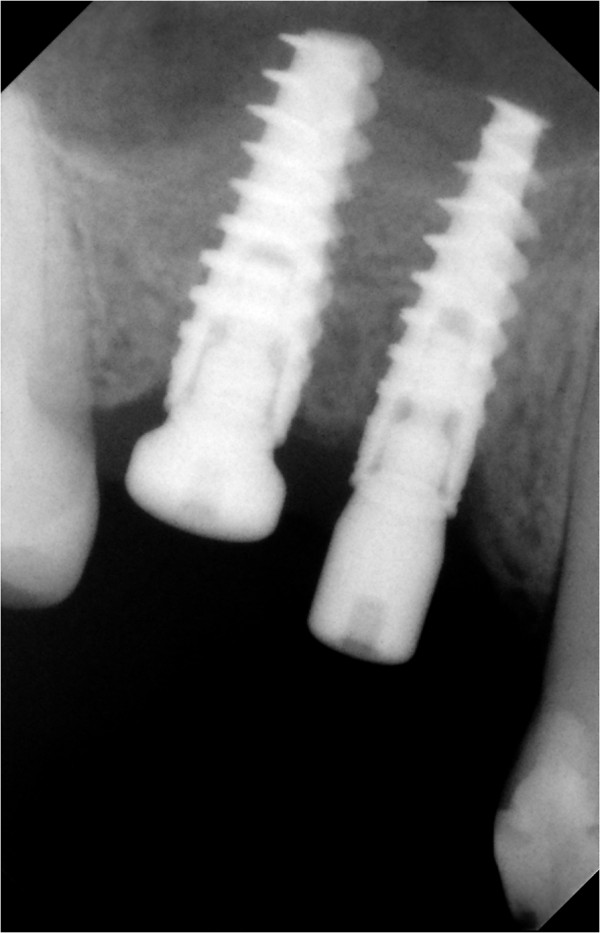
**Radiographic postoperative control at six months.**

The radiographic postoperative control at six months showed a conspicuous neo-bone apposition around the apical area of the implant fixture (Figure 15), in agreement with the indications of Lioubavina-Hack *et al.* [40], according to whom the osteo-implant unit can be considered fully functional when, at X-ray examination, there is an increase in radiopacity surrounding the implant with progressive and gradual decreasing to the periphery.

**Figure 16:**
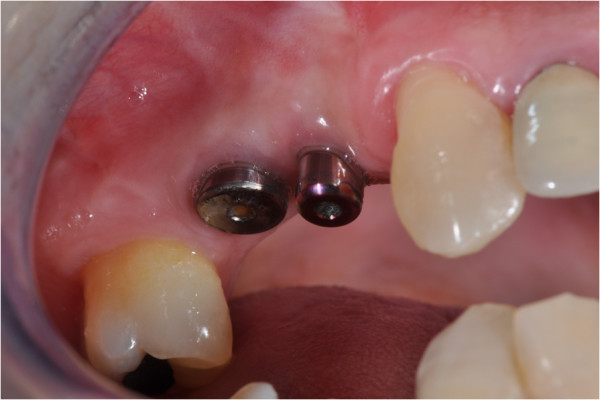
**Clinical aspect of the soft tissue at six months.**

With regard to the condition of the soft tissues, healing took place by first intention, with net retention of keratinized tissue (Figure 16).

To establish the implant survival Albrektsson [41] proposed the following criteria:

1. the system immobility;
2. the absence of peri-implant radiolucency;
3. the absence of persistent inflammation signs or symptoms.

To clinically evaluate the implant stability and osseointegration, we adopted resonance frequency analysis (RFA), a noninvasive diagnostic technique used to determine the ability of an implant to be subjected to load.

RFA is, in fact, the only noninvasive method that can guide the selection of prosthetic times. Adequate implant stability in bone is crucial to allow, after the insertion of an implant, undisturbed healing with new bone formation.

The implant stability is divided into primary and secondary:

– the primary stability is a mechanical parameter that depends on the bone quantity and quality, the system geometry and the adopted technique [42].
– the secondary stability can be considered as the stability increase as a result of the implant placement during the healing.

This increase is attributable to the bone formation and the remodeling process that occurs in the tissue-implant interface in the surrounding area.

For each implant RFA evaluation has been reported as implant stability quotient (ISQ) values. Measurements were performed by an ISQ device (Osstell ISQ, Osstell AB, Gothenburg, Sweden).

Both implants have shown sufficient primary stability associated with mean values of ISQ=54.22.

At the end of the second month, a higher stability than the threshold value of 57 ISQ was recorded in both implants, with an average value of 59.8 ISQ.

A gradual increase in ISQ values was observed until the third and fourth month, reaching an average value of 63.2 ISQ, after which the ISQ values appeared to level off in a straight line.

The gradual filling of the defect is, therefore, accompanied by an increase in the ISQ values (Table 1).

**Table 1:** **Mean implant stability quotient values of the two fixtures from time zero to six months**

Edentulous site	Fixture dimension (mm)	Soft tissues condition	ISQ time 0	ISQ 2 months	ISQ 3 months	ISQ 4 months	ISQ 5 months	ISQ 6 months
**1.4**	3.5 - 13	Good	54	58	61	63	65	65
**1.5**	4.3 - 13	Good	56	61	62	63	64	64

ISQ, implant stability quotient.

**Figure 17:**
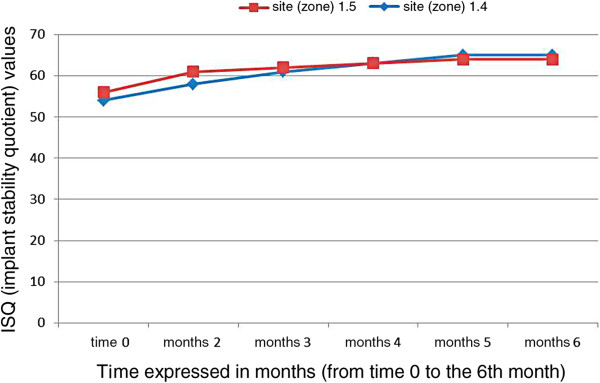
**Performance of implant stability quotient values in the two sites from time zero to six months.** The units of the vertical axis are ISQ (implant stability quotient) values. The unit of the horizontal axis is the time expressed in months (from time 0 to the 6th month).

The ISQ average values trend found in our study (Figure 17) does not seem to be very different from that described in the literature [43] in reports that consider different clinical situations and graft applications, suggesting that the graft incorporation plays a marginal role on implant stability.

On the basis of the recorded values, we can state that the stabilization phase took place between the fourth and sixth month.

## Discussion

Nowadays, blood clot bone regeneration potential is increasingly corroborated by the literature [6, 44]. The present report wants to underline how the blood clot, rich in autogenous growth factors, can act as a natural scaffold between the guide walls of the antral floor, implant screw and raised Schneiderian membrane and differentiate itself into bone-osteoid tissue, providing secondary stability, a necessary condition for osseointegration.

In our study, the Schneiderian membrane lifting created the conditions to allow the filling of the space delimited by its detachment with the stable clot (capable of turning into bone tissue). The space was procured and maintained by the implant placement while the use of a simple equine collagen sponge allowed the clot stabilization in the early stages of healing.

In relation to the results obtained in our study and the related literature, we can state that the grafting materials currently used as subantral space fillers seem to have a more mechanical function that is not purely biological, and this may explain why, in terms of volumetric stability, slow resorption grafts are more effective than autogenous bone [36].

The graftless sinus augmentation technique with contextual implants placement is widely supported by the clinical experience of various authors and also by experimental studies [44, 45].

Srouji *et al.* in 2009 [32], showed how the basal cell layer of Schneider’s membrane has a behavior associated to that of the periosteum, able to produce the osteoprogenitor cells and humoral factors necessary for bone regeneration (BMP2, osteonectin, osteocalcin and osteopontin), requiring only the presence of a stable blood clot.

Lundgren in his report [6], pointed out that the bone deposition, despite the continuous bone remodeling, is the net result of the sinus mucosa elevation in the maxillary sinus augmentation without graft application, while in sites where the technique consisted of bone graft application, a situation of bone resorption predominates. At the end of this article, the author concludes that the simple elevation of the sinus membrane and the simultaneous implant placement resulted in bone formation and osseointegration of implant fixtures.

Many other authors have subsequently observed bone formation after the sinus lift without the use of bone grafts [6, 31, 46–48].

In the context of guided tissue regeneration, various authors have firmly established the importance of blood clot, with its endogenous growth factors, in allowing bone tissue formation [6, 46, 49–54].

The osteogenesis process always starts with the bone defect neovascularization, which is an indispensable condition of every osteogenetic event.

After the colonization of a bone defect by proliferating mesenchymal cells with osteogenic potential capacity, the bone regeneration depends on the influence of the systematically and locally produced bone, by inductor factors such as growth factors and hormones, and by the formation of an appropriate scaffold for the proliferation and differentiation of osteoprogenitor cells.

The first stage is realized during the first four to six weeks.

It is characterized by the formation of a clot and the vascular structures migration by the marrow spaces of the walls that surround the defect, in the space below the membrane, which is followed by the beginning of the osteoid tissue deposition. This tissue is also defined primary spongy tissue and is constituted by bone with interwoven fibers that, advancing, delimit and surround the newly formed vessels, and, merging between them, define the neoformed intertrabecular spaces.

The central part of the defect, not yet filled with regenerated tissue, is composed of loose connective tissue with collagen fibers without orientation, fibroblasts, macrophages, and vases.

The second stage occurs in the subsequent two to three months, during which the maturation of the cancellous bone advances and the formation of cortical bone begins.

The osteoid bone (primary cancellous bone) undergoes mineralization by osteoblast input and at its periphery a new cortical bone, consisting of parallel bundles of lamellar bone, begins the differentiaton.

The lamellar bone is deposited more slowly than the osteoid bone, which has rapidly filled the empty spaces in the first phase, and needs a stable surface on which the collagen fibrils can be deposited in parallel fibers.

The intertrabecular spaces are gradually reduced in volume up to the size of the Haversian canals and, with the neighboring concentric lamellae, they form the primary osteons.

The last phase, which is achieved after three to four months, is characterized by the cortical bone maturation and by the cortical and medullary bone remodeling, a phase that can continue even longer.

In this phase, various osteoclasts invade the remodeling area to eliminate the fibrous bone while the neo-osteoblasts deposit layers of mature lamellar bone that leads to a thinning of the connective tissue.

In the ‘*ex novo*’ bone tissue formation the platelets play a key role during the first phase of the healing process, when there is an initial deposit of fibrin and the formation of blood clot.

This phase is characterized by a significant activation of chemical signals mediated by cytokines and growth factors.

In fact, the posthemorrhagic clot formation process, through platelet aggregation and cell lysis, causes the release of coagulation cascade factors and growth factors, such as platelet-derived growth factor (PDGF), insulin-like growth factors (IGF 1, IGF 2) and vascular endothelial growth factor (VEGF) that are known for their activating effect on osteoblasts and osteoclasts, and transforming growth factor beta (TGF-β), which initiate the formation of bone tissue.

The osteoblastic precursors are responsible, after differentiation into osteoblasts, for the second phase of the healing process (enchondral and/or intramembranous ossification) through the synthesis of collagen and other extracellular matrix components.

A substrate or carrier suitable for the osteoinductive signal is also needed to support and guide the new bone formation. Sampath and Reddi [55] in 1984 have shown that the type I cross-linked collagen is the most appropriate carrier to promote the activity of the osteoinductive signal.

The collagen is needed in the processes of tissue repair for its osteoblastic and angiogenic activity, and also for its hemostatic and debridement properties. The collagen bound to fibronectin promotes the anchoring of mesenchymal stem cells progenitors, which exerts its chemotactic action and allows differentiation into osteoblasts.

Vice versa, through the recruitment of monocytes/macrophages, both osteoblast activity and the angiogenesis process are stimulated at the healing site.

The hemostatic action is exerted as the collagen is able to activate the platelet membrane receptors, responsible for their aggregation and the lysis process. During the first week, the collagen is able to strengthen the fibrin action in the primary clot formation while in the second week it replaces the function of fibrin.

Collagen, also carrying out chemotactic monocytes/macrophages cell lines, promotes the formation of osteoclasts which, through their action in bone resorption, can attract, activate, and collaborate with osteoblasts in bone arrangement and remodeling.

The collagen sponge used in our clinical case offered, therefore, the natural substrate for proper regeneration of bone tissue, facilitating and promoting the physiological process of regeneration. Therefore the space containing the blood clot was created and maintained. This condition allowed the bone regeneration, through the formation of a healing pattern that provided for the migration of cells with an angiogenic and osteogenic potential from the medullary spaces of the adjacent bone tissue to the surgical site. The formation of an angiogenic front and the differentiation of perivascular cells into osteoblasts led to the deposition of extracellular matrix (substantially connective tissue) that was subsequently mineralized in osteoid/bone-like tissue.

The limitations of this technique are represented by clinical situations in which there is not sufficient bone volume in order to ensure the primary stability of the implant fixtures (crestal height <5 to 6mm), and in such cases, it is preferable to insert autologous grafts as fillers instead of synthetic biomaterials, as autologous bone is the gold standard, because it is rich in cytokines, growth factors, and so on, like the blood clot, with the advantage of ensuring an excellent scaffold graft in these circumstances.

On the other hand, the advantages of this technique are: a lower morbidity for the patient, because there is no involvement of a donor site, the lower cost of the procedure, because there is no bone substitutes or membrane application, and the same timing of prosthetic finalization when compared to the techniques that involve the use of bone substitutes.

## Conclusions

Good patient compliance and the motivation for thorough oral hygiene at home has led to an excellent postoperative course. The clinical and radiographic follow-up at 0, 4 and 6 months and the excellent integration of the implant system in the osteo-mucosal context, allowed by proper soft tissue management, has consolidated the success of the surgical technique, *inter alia* with wide confirmation in the literature.

The application of growth factors in grafting biomaterials in order to improve the osteoinductive characteristics increasingly pushes clinicians and researchers to reevaluate the quality of the blood clot, pabulum rich in cytokines and bone morphogenetic proteins, autogenous and able to promote the differentiation bone, with significant biological advantages.

Our radiographic controls showed a continuous and significant peri-implant bone remodeling over time becoming more and more homogeneous.

The results obtained from our study are encouraging and comparable to those achieved with maxillary sinus elevation techniques with biomaterial application.

Our results, therefore, in agreement with those reported in the international literature, allow us to define the graftless sinus augmentation technique as a reliable and predictable surgical method.

## Consent

Written informed consent was obtained from the patient for publication of this case report and any accompanying images. A copy of the written consent is available for review by the Editor-in-Chief of this journal.
